# Possible Error in Reflection Pulse Oximeter Readings as a Result of Applied Pressure

**DOI:** 10.1155/2019/7293813

**Published:** 2019-10-24

**Authors:** Ilya Fine, Alexander Kaminsky

**Affiliations:** Elfi-Tech Ltd., 2 Prof. Bergman St., Science Park, 76705 Rehovot, Israel

## Abstract

Pulse oximetry is one of the most widely used techniques in modern medicine. In pulse oximetry, photoplethysmography (PPG) signals are measured at two different wavelengths and converted into the parameter Gamma, which is used to calculate the oxygen saturation of arterial blood. Although most pulse oximetry sensors are based on transmission geometry, the reflection mode is required for different form factors such as the forehead or wrists. In reflection oximetry, local pressure is applied to the measurement surface. We investigated the relationship between applied pressure and Gamma and found that for the reflection mode, Gamma tends to increase with increasing applied pressure. To explain this, we described the PPG signal in terms of two alternative models: a volumetric model and a Scattering-Driven Model (SDM). We assumed that the application of external pressure results in a decrease in local blood flow. We showed that only SDM correctly qualitatively describes Gamma as a function of the decrease in blood flow. We concluded that both described models coexist and that the relative influence of each depends on the measurement geometry and blood perfusion in the skin.

## 1. Introduction

### 1.1. Alternative PPG Signal Models: Volumetric Model and Scattering Driven Model

Pulse oximetry, which is based on photoplethysmography (PPG), has become the standard technique for noninvasive monitoring of arterial oxygen saturation. Pulse oximetry measures arterial blood hemoglobin saturation (SPO_2_). SPO_2_ is the fraction of oxygen-saturated hemoglobin relative to total hemoglobin [1, 2]. PPG signal is strongly dependent on the absorption of light by hemoglobin which, in turn, depends on the wavelength of the light used. The principle of operation of pulse oximetry is based on the difference in the absorption spectrum of oxy (HbO_2_) and deoxyhemoglobin (Hb) in the visible and infrared spectral regions. HbO_2_ absorbs more light in the near-infrared part of the spectrum (810–990 nm) and lesser light in the red part of the spectrum (630–690 nm). In the most commonly used mode of operation, the light source of a pair of red (670 nm) and infrared (940 nm) light-emitting diodes (LEDs) is used. The measured signals consist of a DC component and pulsatile (AC) component. Pulse oximetry calculates AC/DC at 670 nm and AC/DC at 940 nm. The ratio of these two ratios is called Gamma. Then, empirically derived calibration curves are used to estimate SPO_2_ based on the Gamma. Pulse oximetry technology is robust and successful because the calculated SPO_2_ manifests a very weak dependence on local blood volume, skin pigmentation, hematocrit, and finger size. In practice, two types of geometric configurations of pulse oximeters are widely used. The most common geometry is when the light source shines through the biological tissue, and the photodetector is located on the other side of the measured object. This type of geometry is called transmission pulse oximetry and is typically used on the tip of the finger. With another type of configuration called reflective oximetry, the light sources and detector or detectors are located on one side of the tissue [3]. This configuration is applied on the forehead, on the arm, or other places where it is not possible to use the transmission technique [4].

The PPG signal is commonly associated with changes in local blood volume. It is assumed that the amount of blood in the illuminated perfused tissue fluctuates at the rate of the heartbeat, as does light transmission or refraction. According to this volumetric model, the periodic changes in blood volume result in changes in the intensity of the measured light. Some studies have questioned the uniqueness of the volumetric model for explaining the origin of the PPG signal. For example, Hocherman and Palti [5] simultaneously measured volumetric changes at the fingertip directly, using fluid plethysmography, and optical transmission. They gradually increased the applied pressure on the fingertip until the volumetric signal disappeared; however, the prominent pulsatile optical signal was still observed. In another study, the authors performed Doppler fluorometry and PPG measurements simultaneously in bone [6]. Although a prominent PPG signal was recorded, there was no significant periodic increase in blood volume in bone resulting from vasodilation or the opening of collaterals.

In in vitro studies [7], blood was periodically pumped through a rigid glass cuvette, and a pulsatile optical signal resembling a typical PPG was obtained. In this article, it was shown that such a pulsatile signal is associated with the effect of red blood cell (RBC) aggregation or rouleau formation. Aggregation of red blood cells (RBCs) results in the formation of linear or branched structures called rouleaux [8]. However, in capillary and arterioles, it is reasonable to assume that the development of the branched structures is limited, and linear aggregates prevail. The average length of the RBC aggregates is dependent on shear forces and thus, varies periodically with changes in the blood flow [9, 10]. According to this relationship, the frequency of disassembling and reassembling the aggregates is controlled by the heartbeat, which evokes shear force modulation [11]. These processes are definitely fast enough to result in optical transparency pulsations at the frequency of the heartbeat. Various theoretical approaches have been applied to describe light-scattering process in terms of RBCs size and orientation [12, 13]. Eventually, changes in the length and concentration of scattering particles lead to changes in the scattering of light and accordingly, in the intensity of transmitted light.

Thus, the following question arises: Is it possible to observe the manifestations of RBC aggregation in vivo? Shvartsman and Fine [14] showed that if the blood flow is stopped by applying pressure greater than systolic pressure, then the intensity of the light passing through the fingertip gradually increases. Indeed, in stasis, RBCs spontaneously aggregate to form long linear stacks, which may result in a decrease in light scattering. With respect to pulsatile blood flow, the length of the aggregate depends on the hemodynamic characteristics of the blood in a particular vessel. The rouleaux are easily disrupted by shear forces. The aggregation and disaggregation of RBCs are dynamic processes. This shear-dependent variation is the primary cause of the non-Newtonian behavior of blood in human blood vessels [10]. As blood pressure increases to its peak, the flow velocity and shear rate gradually increase, and as the pressure decreases, the velocity and shear rate decrease. Therefore, the mean size of RBC aggregates is governed by shear forces. As the shear rate increases, the size of the aggregates decreases. Based on these facts, Fine [15] proposed a model that explains the PPG signal in terms of modulation of the scattering of light occurring during the aggregation and disaggregation of red blood cells.

Nevertheless, for a pulsating signal in vivo, there is no direct evidence that the aggregation model can be used as at least an additional phenomenon of the PPG if not as an alternative. The aim of this work was to check (a) whether the aggregation model can correctly describe quantitatively the experimentally known Gamma values used in pulse oximetry and (b) whether Gamma is affected by local blood flow.

## 2. Pulse Oximetry Parameter “Gamma” in Terms of Scattering and Absorption of RBC

### 2.1. Light Intensity as a Function of Scattering and Absorption by RBCs

Diffusion theory for light transmission in tissue is a commonly accepted way to model light propagation in a high-scattering isotropic turbid media such as biological tissue [16, 17]. The key model parameters of effective attenuation coefficient *μ*_eff_^*∗*^(*λ*, *t*) of the blood, or inverse diffusion length, and blood layer thickness *x*(*t*) are used to describe light propagation characteristics through a medium. The intensity of transmitted light (*I*) is a function of these two parameters:

(1) $$\begin{array}{c} I\left(t\right)≅I\left[\mu_{\text{eff}}^{*}\left(t\right),x\left(t\right)\right]. \end{array}$$

where the effective attenuation coefficient for an ensemble of RBCs can be defined by the following approximation [18]:

(2) $$\begin{array}{c} \mu_{\text{eff}}^{*}\left(\lambda ,t\right)=\sqrt{3\;\cdot \;\left[\mu_{a}{\left(\lambda \right)}^{2}+\mu_{a\;}\left(\lambda \right)\cdot \mu_{s}^{\prime }\left(\lambda ,t\right)\right]}, \end{array}$$

where *μ*_*a*_(*λ*) is the absorption coefficient and *μ*_*s*_′(*λ*, *t*)=*μ*_*s*_ (*λ*, *t*) · [1 − *g*(*λ*, *t*)], where *μ*_*s*_ (*λ*, *t*) is the scattering coefficient, and *g* is the scattering anisotropy.

The light absorbance of RBCs is a function of oxyhemoglobin and deoxyhemoglobin. Oxygen saturation SPO_2_ is defined as the ratio of the concentration of oxyhemoglobin [HbO_2_] and that of deoxyhemoglobin [Hb] plus [HbO_2_]:

(3) $$\begin{array}{c} {\text{SPO}}_{2}=\frac{\left[{\text{HbO}}_{2}\right]}{\left[{\text{HbO}}_{2}\right]+\left[\text{Hb}\right]} \end{array}$$

The average absorption coefficient is given by the following expression:

(4) $$\begin{array}{c} \mu_{a}\left(\lambda \right)=\left[{\text{SPO}}_{2}\cdot \sigma_{{\text{HbO}}_{2}\left(\lambda \right)}+\left(1-{\text{SPO}}_{2}\right)\cdot \sigma_{\text{Hb}\left(\lambda \right)}\right]\cdot \rho , \end{array}$$

where *σ*_HbO_2_(*λ*)_ and *σ*_Hb(*λ*)_ are the absorption cross sections of oxy- and deoxyhemoglobin, respectively, and *ρ* is the concentration of erythrocytes particles and is defined as *ρ*=*H*/*V*, where *V* is the volume of the particles and *H* is the hematocrit value. We can assume that arterial blood is almost entirely oxygenated, so *μ*_*a*_(*λ*) ≈ *μ*_HbO_2_(*λ*)_. For radiation propagating through a statistically homogeneous random medium, the attenuation as a function of the optical length is close to exponential [19]. The validity of the exponential approximation for light transmission in perfused tissue is also fortified by the fact that the Gamma used in pulse oximetry is independent of the amount of blood. Only the exponential dependence of light transmission on the thickness of blood removes the dependence of Gamma on the thickness of the finger. This is the basis of pulse oximetry. Therefore, we assumed that the degree of attenuation of the transmitted light intensity in the medium in which the tissue is penetrated by blood vessels is determined by the following exponential expression:

(5) $$\begin{array}{c} I\left(t\right)∼\;\exp\;\left[-\mu_{\text{eff}}^{*}\left(t\right)\cdot x\left(t\right)\right]. \end{array}$$

The exponential dependence of *I*(*t*) is predetermined by the diffusion properties of the surrounding tissue. Thus, it is not paradoxical that the pulse oximeter functions correctly only because of the presence of light-scattering tissue. The scattering coefficients of the blood volume can be expressed in terms of the scattering cross section and RBC concentration *ρ*(*t*):

(6) $$\begin{array}{c} \mu_{s}\left(\lambda ,t\right)=\sigma_{s}\left(\lambda ,t\right)\cdot P\cdot \rho \left(t\right), \end{array}$$

where *σ*_*s*_(*λ*, *t*) is the scattering cross section, and *P* is the packing factor, initially introduced by Twersky [20]; it ranges from 0.2 to 0.65. For a suspension of single RBCs, P=(1 − H)(1.4 − H). Thus, *μ*_*s*_′(*λ*, *t*)=*σ*_*s*_(*λ*, *t*) · *P* · *ρ*(*t*)  · [1 − *g*(*λ*, *t*)].

The PPG signal is characterized by changes in the intensity of the light after it passes through blood and tissue. The associated pressure waves give rise to periodic changes in the optical properties of the measured blood vessels. However, we are interested in the specific mechanism that causes the changes in the optical properties of the perfused tissue being measured. We considered two different mechanisms that may be responsible for the changes in light intensity as a function of time. One mechanism is called the volumetric model. This is the most accepted model of PPG, whereby during the systolic phase, a pressure wave leads to an increase in blood volume in the tissue. Thus, in equation (1), only *x* is a function of time.

The second, alternative model is known as the scattering-driven model (SDM) whereby the blood pressure wave induces changes in the light-scattering characteristics of blood, most likely through the RBC aggregation-disaggregation mechanism. The changes in RBC aggregation are driven by variations in the shear rate. Formally, we assume that just *μ*_*s*_(*λ*, *t*) depends on time. Periodic changes in speed can lead not only to changes in RBC aggregation but also to a change in the orientation of the RBCs or the density of RBC distribution in the vessels [10]. All these phenomena are associated with a change in light scattering.

**Figure 1:**
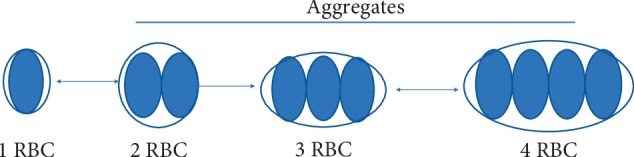
Aggregation of RBCs is approximated by an ellipsoid as the number of RBCs increases.

To describe the light scattering by RBCs, we used the Mie [21] solution, which provides a complete solution for determining the scattering cross section value and the phase-scattering function. Steinke and Shepherd [22] showed that the Mie model can be used to describe light scattering by RBCs. To adjust the Mie theory for use with RBC aggregates, we used the following simplified model: when RBCs stick together in a chain, the new scattering entity can be approximated as a spheroid (Figure 1). For short aggregates, the spheroid is flattened, while it becomes elongated with an increase in the number of particles.

**Figure 2:**
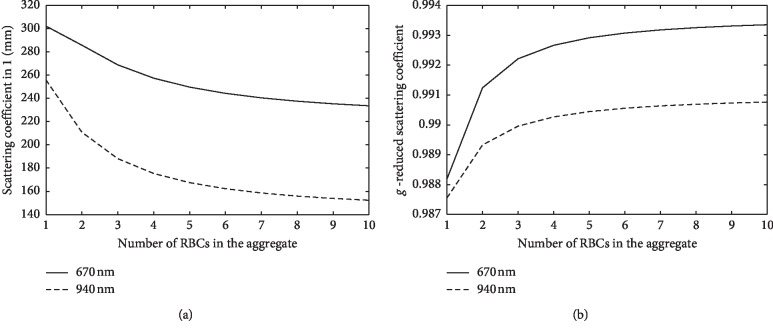
(a) Scattering coefficient as a function of number of RBCs in the aggregate and (b) reduced scattering as a function of number of RBCs in the aggregate.

According to the Mie model, to calculate the scattering cross section, the radius of the sphere, *r*, and relative refractive index are required. To circumvent this problem, we used an ellipsoid with a revolution of a given size, axial ratio, and orientation that could be approximated by a suitable equivalent sphere [23]. The effects of the difference between the volumes of the ellipsoid and the equivalent sphere can be accounted for using a corrected refractive index *m*_*c*_ for the equivalent sphere:

(7) $$\begin{array}{c} m_{c}=1+\left(m-1\right)\cdot \chi \left(\Omega \right), \end{array}$$

where *m* is the refractive index of the particle relative to the surroundings, *χ*(*Ω*) can be expressed in terms of axial ratio parameters of the ellipsoid and the angle (*Ω*) of incidence of light relative to the main axis of the ellipsoid. We assumed that the incident light interacts with an ensemble of randomly oriented RBC aggregates, so the scattering cross section and *χ*(*Ω*) should be averaged over all possible directions by using equation (7). Therefore, we calculated the mean values of *μ*_*s*_ and *g* using the adjusted Mie equations for an ellipsoid composed of *n* RBCs (*n* = 1–10) (Figure 2).

### 2.2. Expressions for Gamma

In pulse oximetry, the value of SPO_2_ is determined entirely by Gamma, which is defined as the ratio of the pulsatile (AC) and nonpulsatile (*I*) components of the red and infrared signals and is known as the ratio of ratios:

(8) $$\begin{array}{c} \text{Gamma}=\frac{\text{delta }\left[I\left(\lambda_{1},t\right)\right]/I\left(\left(\lambda_{1},t\right)\right.}{\text{delta }\left[I\left(\lambda_{2},t\right)\right]/I\left(\lambda_{2},t\right)}, \end{array}$$

where delta (*I*) is associated with the AC component of the signal. AC represents the amplitude of the PPG signal. For small changes in AC, Gamma can be approximated by the so-called parametric slope, given by

(9) $$\begin{array}{c} \text{Gamma}=\frac{\partial \;\ln\;\left[I\left(\lambda_{1},t\right)\right]/\partial t}{\partial \;\ln\;\left[I\left(\lambda_{2},t\right)\right]/\partial t}. \end{array}$$

Next, we called Gamma for the SDM as GammaS and Gamma for the volumetric model as GammaV. The important property of Gamma is that its value is practically unaffected by the local blood volume, blood hematocrit, measurement geometry, and tissue hematocrit. This implies that Gamma has the striking feature of an invariant that depends upon absorption and scattering properties only. Gamma can be converted into SPO_2_ using an experimentally obtained calibration curve.

To derive an explicit expression for Gamma, we used equations (2)−(5), (7), and (9). In the most general case,

(10) $$\begin{array}{c} \text{Gamma}=\frac{\partial \;\ln\;I\left[\mu_{s}^{\prime }\left(\lambda_{1},t\right),\mu_{a}\left(\lambda_{1}\right),x\left(t\right)\right]/\partial t}{\partial \;\ln\;I\left[\mu_{s}^{\prime }\left(\lambda_{2},t\right),\mu_{a}\left(\lambda_{2}\right),x\left(t\right)\right]/\partial t}. \end{array}$$

Using equation (10), we calculated Gamma as a function of the size of the rouleaux for the volumetric model and the SDM. The only time-dependent parameter for the volumetric model is *x*:

(11) $$\begin{array}{c} \text{GammaV}=\frac{\partial \;\ln\;I\left[\mu_{s}^{\prime }\left(\lambda_{1}\right),\mu_{a}\left(\lambda_{1}\right),x\left(t\right)\right]/\partial x}{\partial \;\ln\;I\left[\mu_{s}^{\prime }\left(\lambda_{2}\right),\mu_{a}\left(\lambda_{2}\right),x\left(t\right)\right]/\partial x}. \end{array}$$

For the SDM model, *x* is fixed, and *μ*_*s*_′ and particle concentration *ρ* are functions of time:

(12) $$\begin{array}{c} \text{GammaS}=\frac{\partial \;\ln\;I\left[\mu_{s}^{\prime }\left(\lambda_{1},t\right),\mu_{a}\left(\lambda_{1}\right),x\right]/\partial \mu_{s}^{\prime }\left(\lambda_{1}\right)}{\partial \;\ln\;I\left[\mu_{s}^{\prime }\left(\lambda_{2},t\right),\mu_{a}\left(\lambda_{2}\right),x\right]/\partial \mu_{s}^{\prime }\left(\lambda_{2}\right)}\;\times \;\frac{\partial \mu_{s}^{\prime }\left(\lambda_{1},t\right)/\partial t}{\partial \mu_{s}^{\prime }\left(\lambda_{2},t\right)/\partial t}. \end{array}$$

**Figure 3:**
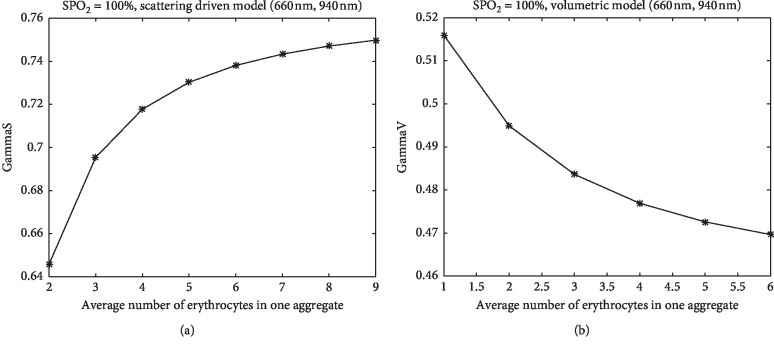
Gamma dependence on RBC aggregation length for (a) SDM and (b) volumetric model.

To calculate Gamma, we chose commonly used wavelengths of 660 and 940 nm and SPO_2_ = 100%, which approximately corresponds to the normal level of oxygenation of arterial blood. Figures 3(a) and 3(b) show the estimated GammaS and GammaV as a function of the average size of the RBC aggregates. Gamma gradually increases with increasing aggregate size, whereas for the volumetric model, GammaV has a weak dependence on the length of the aggregate. For one RBC or a small rouleau, the difference between GammaS and GammaV is small, and their values are close to the range of the experimental values known in pulse oximetry.

## 3. Experimental Results and Discussion

### 3.1. Experimental System

The goal of our study was to examine experimentally the behavior of Gamma for two types of measurement geometry: reflection and transmission. The idea was to create conditions under which the average length of RBC aggregates could be increased by changing the local blood flow. A decrease in blood flow velocity should lead to a shift in dynamic equilibrium upon which the average length of the aggregates should increase. We reduced the blood flow velocity by applying pressure. In this way, we investigated the dependence of Gamma on the level of local pressure for several form factors: fingertip (transmission and reflection), wrist, and forehead.

**Figure 4:**
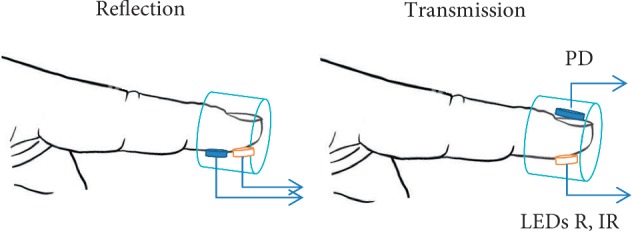
Measurement setup for reflection and transmission.

For pulse oximetry, we used a standard optical system that consisted of two LEDs at 660 and 940 nm and a photodetector (PD) with an amplifier. The digitized signal was stored in a computer for further processing and analysis. Inflatable silicone cushions were used to create local pressure (Figure 4).

**Figure 5:**
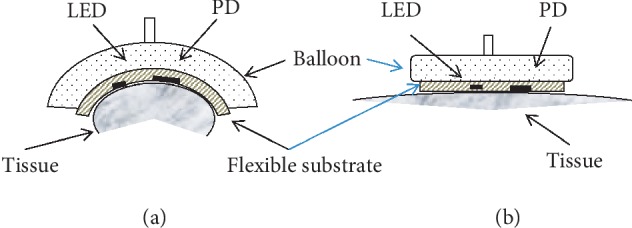
(a) Curved surface of the tissue (finger). (b) Flat surface of the tissue (forehead).

The pressure level in the cushions was set using a pressure controller, which was managed by software on the computer. The difference between the pressure in the cushion and the pressure applied to the skin was taken into account [24]. In our case, the experimentally determined pressure transfer function was defined as P _Skin_=0.92 · P _Balloon_ − 20 [torr]. In the reflection mode, the LEDs and photodiode (PD) are mounted side-by-side on the same planar substrate. A flexible substrate was used to adjust the shape of the tissue (Figure 5).

### 3.2. Results

**Figure 6:**
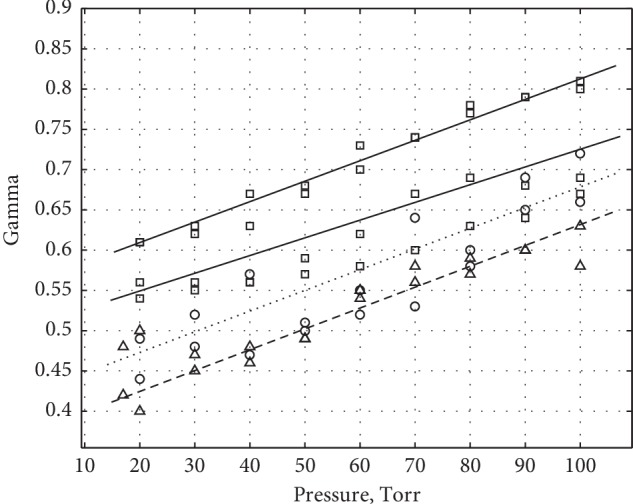
Gamma as a function of applied pressure at different locations for reflection geometry. Squares: fingertip; triangles: forehead; circles: wrist. Filled and open symbols indicate the two sets of measurements taken at the different locations.

Figure 6 shows the experimental data for Gamma, as a function of applied pressure for reflection geometry measured at three different locations: the finger, the wrist, and the forehead. The Gamma was calculated by using equation (8). Each point on the graph was obtained by averaging several measurements. In all cases, Gamma tended to increase with increasing pressure.

**Figure 7:**
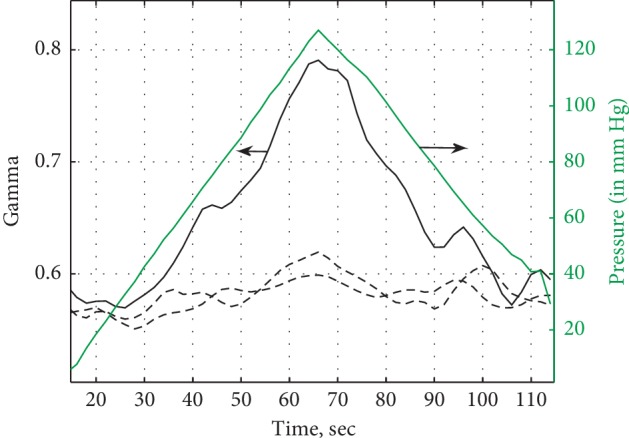
Gamma as a function of applied pressure on the finger for transmission (dashed lines) and reflection (solid line) geometry. Right ordinate axis is the applied pressure, and the green line is the pressure as a function of time (in torr).

Figure 7 shows the behavior of Gamma for transmission geometry and reflection geometry. The cuff pressure was gradually increased to 120 torr and then gradually decreased to the initial pressure. In most cases, the change in Gamma for transmission geometry was very subtle.

### 3.3. Discussion

We analyzed different possible causes of the dependence of Gamma on the pressure applied. For reflection geometry, one can speculate that applied pressure may induce the so-called crosstalk effect. In other words, the external pressure applied to the tissue “squeezes” the blood out of the capillaries of the dermis, which leads to direct leakage of the specular component of light from the LED to the detector. To test this assumption, we made the following assessment: assume that the increase in the baseline of the PPG signal (DC component) as the pressure increases is entirely due to crosstalk. In this case, the effect of crosstalk on the Gamma value should be maximal. To obtain the measured Gamma value caused by the alleged crosstalk, we substituted the experimentally measured change in the DC for the red (*δI*_*R*_) and infrared (*δI*_*IR*_) channels into the following expression:

(13) $$\begin{array}{c} \text{Gamma Crosstalk}=\text{Gamma}\cdot \left(\frac{I_{R}}{I_{IR}}\right)\cdot \left(\frac{I_{IR}+\delta I_{IR}}{I_{R}+\delta I_{R}}\right). \end{array}$$

These changes are due to the applied pressure. We found that the measured changes in Gamma were significantly greater than those estimated by equation (13). Thus, the crosstalk effect is not sufficient to explain the results. To reaffirm this conclusion, we conducted experiments using different PPG sensor geometries by using bases (distances) of *L* = 4 mm and *L* = 12 mm between the LED and the PD. The crosstalk effect for *L* = 12 mm was qualitatively the same as that for *L* = 4 mm, and the crosstalk effect for *L* = 12 mm was supposed to be negligible. Thus, for reflection geometry, we concluded that applied pressure has an effect on Gamma regardless of the sensor geometry.

We explained our experimental results for reflection geometry by assuming that the applied pressure results in a decrease in blood flow velocity and shear rate in the arteriole vessels. Following this process, the dynamic balance between the formation and the destruction of aggregates shifts toward longer aggregates. According to the prediction of the SDM, Gamma should increase, as was observed in our experiments.

However, a similar effect is not observed in transmission geometry. This is explained by the significant connection between vessel diameter and shear rate forces. Relatively large arteries with diameters between 150 and 242 *μ*m are located at the hypodermal-dermal junction [25]. The arterioles in the papillary dermis vary from 17 to 26 *μ*m in diameter and represent terminal arterioles. They are located at the very “bottom” of the dermis. The arterioles and venules involved in cutaneous microcirculation form two important plexuses in the dermis: an upper horizontal network from which the nutritive capillary loops of the dermal papillae arise and a lower horizontal network at the dermal-subcutaneous interface. Therefore, in reflection geometry, most of the signal derives from the tiny vessels where the dependence on shear rate is very strong. In transmission geometry, light passes through deeper layers where the diameters of the vessels are much larger and, therefore, initially, the change in the shear forces is much less when pressure is applied. Besides, in wider vessels, the emergence of a volumetric component is much more likely, which is why a significant difference in Gamma between the transmission and volumetric options is observed.

It should be noted that the Gamma values were calculated completely based on the basic properties of red blood cells, including their size and aggregability. It is noted in the literature that the readings of pulse oximeters for sickle-shaped anemia patients, in which both the form of red blood cells and the ability to aggregate are distorted, give systematic deviations [26]. This fact can be explained in the framework of the proposed model. Moreover, the proposed model predicts that pathological changes that significantly affect the scattering properties of red blood cells either through their geometry or by changing the optical properties of blood plasma can lead to errors in the display of pulse oximetry.

## 4. Conclusion

In summary, we showed the dependence of Gamma on the pressure applied to blood vessels in pulse oximetry. The experimental result was explained by applying a model in which the pulsatile changes in the intensity of the reflected light result from the variations in the average size of RBC aggregates. The average aggregate size at each instant of time depends on the size of the vessel and the speed of blood flow. Both volumetric and aggregation models of the PPG signal yield similar Gamma values for small aggregates. With increasing aggregate length, the behavior of Gamma differs between the two models. Therefore, we assumed that the two underlying mechanisms of the PPG signal are superimposed. The relative contribution of each of these mechanisms may depend on the form factor, measurement geometry, and blood flow conditions.

## Data Availability

The raw PPG data used in this study are available from the corresponding author upon request.
